# Aspiration with or without lavage in the treatment of acute suppurative thyroiditis secondary to pyriform sinus fistula

**DOI:** 10.20945/2359-3997000000207

**Published:** 2020-03-18

**Authors:** Huijie Yang, De Li, Xinhua Ye, Jinluo Cheng, Zhongzhi Jia, Xuechun Huang, Xiang Wang, Ying Xu

**Affiliations:** 1 Department of Endocrinology Affiliated Changzhou No. 2 People’s Hospital Nanjing Medical University Changzhou Jiangsu China Department of Endocrinology , The Affiliated Changzhou No. 2 People’s Hospital of Nanjing Medical University , Changzhou , Jiangsu , China; 2 Department of Interventional Radiology Affiliated Changzhou No. 2 People’s Hospital Nanjing Medical University Changzhou Jiangsu China Department of Interventional Radiology , The Affiliated Changzhou No. 2 People’s Hospital of Nanjing Medical University , Changzhou , Jiangsu , China; 3 Department of Ultrasound Affiliated Changzhou No. 2 People’s Hospital Nanjing Medical University Changzhou Jiangsu China Department of Ultrasound , The Affiliated Changzhou No. 2 People’s Hospital of Nanjing Medical University , Changzhou , Jiangsu , China; 4 Department of Radiology Affiliated Changzhou No. 2 People’s Hospital Nanjing Medical University Changzhou Jiangsu China Department of Radiology , The Affiliated Changzhou No. 2 People’s Hospital of Nanjing Medical University , Changzhou , Jiangsu , China; 5 Department of Otolaryngology Affiliated Changzhou No. 2 People’s Hospital Nanjing Medical University Changzhou Jiangsu China Department of Otolaryngology , The Affiliated Changzhou No. 2 People’s Hospital of Nanjing Medical University , Changzhou , Jiangsu , China

**Keywords:** Acute suppurative thyroiditis, pyriform sinus fistula, management, aspiration, lavage

## Abstract

**Objective:**

There is currently no consensus regarding the optimal management of acute suppurative thyroiditis (AST) secondary to pyriform sinus fistula (PSF). To investigate the benefits and adverse events of aspiration with or without lavage for the treatment of AST secondary to PSF.

**Subjects and methods:**

This was a retrospective analysis of consecutive patients with AST secondary to PSF who were admitted at the Affiliated Changzhou No. 2 People’s Hospital of Nanjing Medical University between August 2012 and December 2018. Clinical information, procedural data, and imaging data were analyzed.

**Results:**

Seven patients (five women; mean age, 16.9 ± 6.3 years; range, 8-26 years) were included. The patients most presented with anterior neck pain and swelling (n = 7), fever (n = 7), or odynophagia (n = 5). Six cases of AST occurred on the left side of the thyroid and 1 on the right. All patients had thyroid abscess. AST was diagnosed by ultrasound-guided needle aspiration cytology in all cases. PSF was diagnosed during the inflammatory stage in five patients and during the quiescent stage in two. All patients were treated with empiric antibiotics. Needle aspiration without lavage was performed in three cases. Needle aspiration with lavage was performed in four cases. Repeat aspiration was performed in three cases. All patients recovered completely, with no procedure-related complications. During 18.3 ± 7.8 months of follow-up, AST recurred in one case. Excision of the PSF was performed in another case.

**Conclusion:**

Ultrasound-guided aspiration with or without lavage had a good treatment effect and without adverse events for the management of AST secondary to PSF.

## INTRODUCTION

Acute suppurative thyroiditis (AST) is an extremely rare disease representing 0.1-0.7% of all cases of thyroid diseases ( 1 - 3 ). Infectious thyroiditis is uncommon because the thyroid gland is a completely encapsulated structure with sufficient blood supply, extensive lymphatic drainage, and a high concentration of iodine ( 4 ). It occurs mainly in children with congenital pyriform sinus fistula (PSF) ( 4 , 5 ); only rarely has this disease been reported among adults with PSF ( 6 , 7 ), without detectable PSF ( 3 ), after a thyroid diagnostic procedure ( 8 ), or by foreign bodies like fish bones ( 8 , 9 ). AST may lead to abscess, concurrent suppurative mediastinitis, tracheal or esophageal fistula, or even pressure on the trachea leading to asphyxiation ( 10 ). Onset is sudden and the disease progresses rapidly ( 11 ). Thyrotoxicosis has been reported in some cases ( 8 ).

Several treatment options are available for the management of AST secondary to PSF, including conservative treatment with an antibiotic alone, incision and drainage, and open surgery combined with partial thyroidectomy ( 2 ). Antibiotic alone can only be used for early AST. Incision and drainage and open surgery are expensive and are associated with surgical trauma ( 8 ). Catheter drainage has been suggested as a less invasive alternative than surgical drainage ( 9 , 11 ). Injecting an antibiotic into the abscess after pus drainage is also an alternative ( 9 ). Yeow and cols. ( 12 ) recommend needle aspiration for lesions < 3 cm and catheter drainage for lesions > 3 cm. About 20% of patients who undergo drainage will require surgery ( 13 ). Nevertheless, there is currently no consensus regarding the optimal management of AST secondary to PSF.

In this retrospective study, we sought to investigate the benefits and adverse events of aspiration with or without lavage for the treatment of AST secondary to PSF. We also propose a potential treatment algorithm for AST and diagnostic method for PSF based on the results of this study and the literature.

## SUBJECTS AND METHODS

This was a retrospective analysis of consecutive patients with AST secondary to PSF who were admitted at the Affiliated Changzhou No. 2 People’s Hospital of Nanjing Medical University between August 2012 and September 2018. The cases were identified through departmental procedural logs. Patient demographics, clinical information, and procedural data were extracted from the patients’ medical records. This study was approved by the institutional review board of the Affiliated Changzhou No. 2 People’s Hospital of Nanjing Medical University, with a waiver regarding informed consent.

### Diagnosis of AST and PSF

Patients with suspected AST (presenting with anterior neck swelling, pain, and fever) underwent blood routine, infectious index, thyroid function, ultrasound (US), US-guided aspiration cytology, and pus bacterial culture. Imaging examinations, including esophagram, computed tomography (CT), magnetic resonance imaging (MRI), and/or laryngoscopy were performed in all patients to find the PSF.

AST was diagnosed with a positive pus bacterial culture. It could also be diagnosed with a combination of increased white blood cell levels (>10×10 ^9^ /L), abnormal ultrasound (unclear hypoechoic area or mixed echo lesion with heterogeneous content), and an abundance of inflammatory cells on cytology.

PSF can be diagnosed as follows: 1) presence of intraoral pus or feeling of saline in throat during lavage or feeling of methylene blue or contrast agent in throat after injection into the abscess; 2) fistula originating from the apex of the pyriform recess on esophagram; 3) fistula tract filled with barium or meglumine diatrizoate on CT; 4) finding of PSF on MRI; 5) fistula orifice on laryngoscopy; or 6) meglumine diatrizoate diffusion into the pyriform sinus on CT scan when agent was injected into the abscess. The inflammatory stage was defined as occurrence of AST and the quiescent stage was defined as the complete elimination of inflammation, namely after 3 months of follow-up.

### Treatment of AST

When AST was diagnosed, intravenous antibiotic therapy was routinely initiated in all patients. The choice of antibiotic was based on availability but generally included coverage of Gram-positive coccus organisms. If available, the choice was based on blood or pus culture results.

Aspiration was performed by the same physician without anesthesia when the diagnosis of AST was established and an abscess was found by US. A 20-gauge intravenous catheter was inserted into the abscess under US guidance. The content was aspirated with a 10-mL syringe and the lesion was rinsed by injecting normal saline. This process was repeated until further removal of pus was not possible and the liquid turned to bloody. US was performed every day. If pus reappeared, repeated aspiration was carried out.

**Figure 1:**
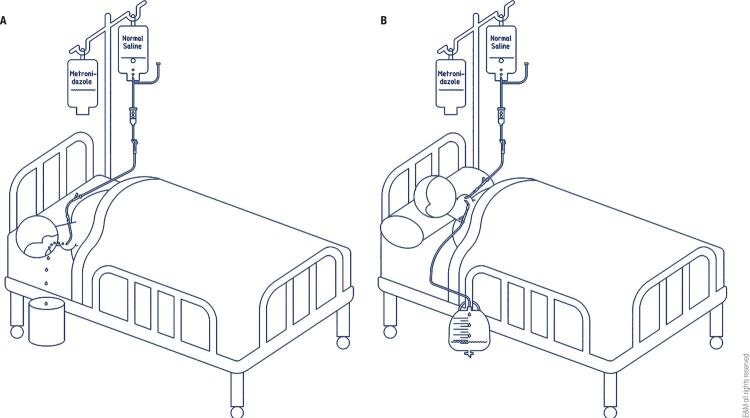
Lavage performed using normal saline and metronidazole via a 20-gauge catheter. (a) Normal saline and metronidazole drained via the PSF. (b) Normal saline and metronidazole drained via another catheter.

Lavage was performed when a liquefied abscess (well-formed abscess as opposed to phlegmon) was found using US. Adequate pressure was maintained in the abscess cavity during rinsing and trying to open the fistula channel using patient’s pharyngeal perception of saline taste and flushing fluid could be spilled from the mouth. Then, normal saline (1000 mL) was injected into the abscess cavity via an intravenous catheter at 15-20 mL/min, one time per day and was drained to the mouth via the PSF ( Figure 1A ). If the solution did not penetrate the fistula, the abscess was repunctured with another 20 gauge intravenous catheter; the two catheters were indwelled and fixed into the pus cavity. Normal saline (1000 mL) at 15-20 mL/min, two times per day, was injected into the abscess cavity via one catheter and drained by the other ( Figure 1B ). Metronidazole (0.5 g) was administered via the catheter immediately after administration of the normal saline. The lavage was stopped when no abscess was drained out and the drainage turned to bloody.

Resolution of AST was defined as resolution of the associated clinical symptoms and resolution of the need for a percutaneous drain or intravenous antibiotics, which was judged comprehensively through clinical manifestations, US, blood routine examination, and infectious indicators.

### Clinical follow-up

After discharge, outpatient clinic visits were offered on months 1, 3, 6, and 12. At each visit, the patients were asked whether the symptoms of AST recurred and inspection and palpation of the anterior neck were performed. Thyroid function (thyroid stimulating hormone, free triiodothyronine, and free thyroxine) and US results were assessed at 1 month. Imaging with esophagram, laryngoscopy, CT, and/or MRI was performed on the third or sixth month. After 1 year, routine follow-up was not needed.

## RESULTS

### Patients characteristics

From August 2012 to September 2018, seven patients (two men, five women; mean age, 16.9 ± 6.3 years; range, 8-26 years) were diagnosed with AST. All of these cases were secondary to PSF: six with left PSF and one with right PSF. All patients presented with anterior neck pain and swelling with or without odynophagia, fever, dysphonia, and headache. All patients had been misdiagnosed. All patients had abscess formation (Supplementary Table 1).

### Diagnosis of AST and PSF

**Figure 2:**
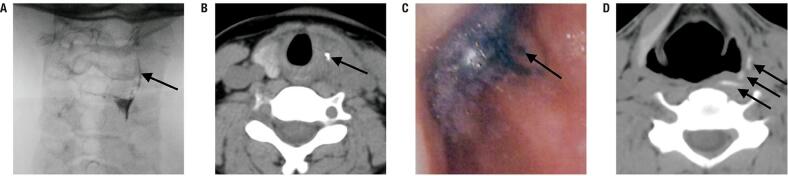
Diagnosis of PSF during the inflammatory stage. (A) Esophagram showing a fistula originating from the apex of the pyriform recess, and (B) CT scan showing the fistula filled with barium after esophagram (case 1). (C) Mythylene injected into the thyroid abscess and found by laryngoscopy (case 3). (D) CT scan performed 2 h after injection of meglumine diatrzoate into the thyroid abscess showing contrast agent infused into the piriform sinus (case 4).

**Figure 3:**
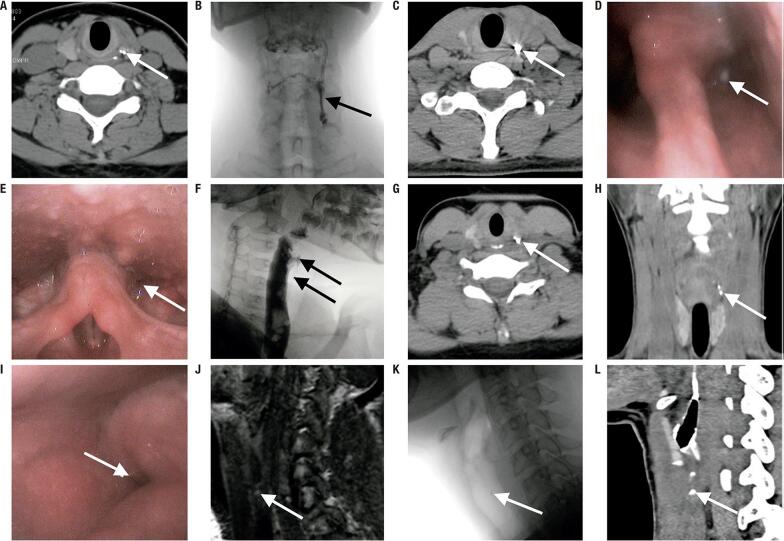
Diagnosis and follow-up of PSF during the quiescent stage. (A) CT revealing fine fistula filled with meglumine diatrzoate after oral administration (case 2). The results from esophagram, laryngoscopy, and MRI were all negative (not shown). (B) Esophagram(oral barium) showing a PSF; (C) CT after esophagram showing the fistula filled with barium; and (D) laryngoscopy showing the fistula orifice stained with barium (case 4). (E) Laryngoscopy demonstrating the fistula orifice; (F) esophagram (oral barium) revealing the fistula originating from the apex of the pyriform recess and extending to the level of c4/5; and (G) CT after esophagram showing that the PSF was filled with barium (case 5). (H) CT after esophagram (oral barium) showing fine PSF filled with barium (case 6), but the esophagram was negative and the laryngoscopy was suspicious (not shown). (I) laryngoscopy showing the suspicious right fistula orifice and the left side normal; (J) T2WI shows the high signal dot shadow at the lower edge of the right piriform sinus; (K) esophagram (oral meglumine diatrizoate) revealing a suspicious slightly high signal dot shadow at the level of C6 at the right neck; (L) CT after oral meglumine diatrizoate showing the obvious PSF and the position consistent with esophagram, MRI (case 7).

All AST cases were diagnosed by US and US-guided needle aspiration cytology. Blood and pus cultures were performed in four and seven patients, respectively. All blood cultures were negative, whereas pus cultures were positive in four cases. The mean interval between the onset of symptoms and initial diagnosis of AST was 12.9 ± 7.3 days (range, 4-21 days) (Supplementary Table 1). PSF was diagnosed during the inflammatory stage in five patients (cases 1, 2, 3, 4, and 7) and during the quiescent stage in two patients (cases 5 and 6) ( Table 1 , Figures 2 and 3 ).

**Table 1:** Diagnosis of PSF during the inflammatory and/or quiescent stages

Case	Stage	Clinical Diagnosis	CT	MRI	Esophagram	Laryngoscopy
1 ^a^	Inflammatory	NP	Positive	NP	Positive	NP
	Quiescent		Positive	NP	Positive	NP
2	Inflammatory Quiescent	Positive	Negative Positive	Negative Negative	NP Negative	Negative Negative
3	Inflammatory	Positive	Negative	NP	NP	Positive
	Quiescent		Negative	Negative	Negative	Negative
4	Inflammatory Quiescent	Positive	Positive Positive	Negative NP	NP Positive	Negative Positive
5	Inflammatory	Negative	Negative	NP	NP	NP
	Quiescent		Positive	Negative	Positive	Positive
6	Inflammatory	Negative	NP	NP	NP	NP
	Quiescent		Positive	NP	Negative	Suspicious
7	Inflammatory Quiescent	Positive	Negative Positive	Negative Positive	Negative Suspicious	Suspicious Suspicious

CT: computed tomography; MRI: magnetic resonance imaging; NP: not performed; PSF: pyriform sinus fistula.

^a^ This patient underwent fistulectomy during the quiescent stage of PSF.

### Treatment and outcomes

All patients were treated with empiric antibiotic therapy and needle aspiration. Repeated aspiration had to be carried out in three patients because of abscess recurrence after initial aspiration (cases 2, 3, and 7). Lavage was performed in cases 2, 3, 4, and 7 because of complete liquefaction of the abscess. Lavage was performed once in cases 2, 3, and 7, and six times in case 4. The normal saline was drained via the PSF in cases 2, 3, and 7, and by a second catheter in case 4. After aspiration and lavage, all patients’ anterior neck pain and swelling eased off quickly.

**Figure 4:**
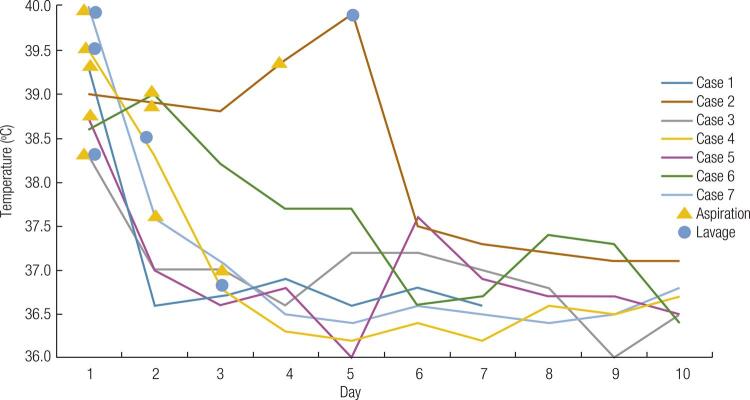
Body temperature changes of six study patients during hospitalization.

Body temperature dropped rapidly during hospitalization for all seven patients ( Figure 4 ). All patients recovered completely and there were no complications related to the aspiration or lavage procedures.

During a mean 18.3 ± 7.8 months of follow-up, thyroid function tests and thyroid US scans were normal. AST did recur 9 months later in case 5. Thanks to the provided health education, this patient consulted 2 days after recurrence and recovered completely after treatment with intravenous antibiotics in the very early inflammation Treatment for the PSF was recommended but refused due to financial reasons. Case 1 underwent PSF excision 1 month after discharge, with no postoperative complications. The remaining five patients received no additional treatment for PSF, but had no recurrence.

## DISCUSSION

There is currently no consensus regarding the optimal management of AST secondary to PSF. This study aimed to investigate the benefits and adverse events of aspiration with or without lavage for the treatment of AST secondary to PSF. The results suggest that US-guided aspiration with or without lavage had a good treatment effect and without adverse events for the management of AST secondary to PSF.

AST occurs mainly in patients with anatomical deformities such as PSF or thyroglossal duct cyst ( 14 ). AST also occurs occasionally in patients with diseases such as goiter, malignancies ( 15 , 16 ), and immunodeficiency ( 17 ), and occurs rarely in patients undergoing fine needle aspiration ( 8 ) or with a history of trauma ( 18 , 19 ). The pathogens that most commonly cause AST are Gram-positive cocci that can colonize the oral mucosa and spread to the thyroid. These pathogens are usually identified as Staphylococcus and Streptococcus species, but other pathogens have occasionally been identified, including Salmonella species, Mycobacterium species, and mixed flora ( 2 , 20 ). In the present study, all cases of AST were secondary to PSF and the pathogenic causes of AST were identified as Streptococcus species in four patients.

Several therapies are used for the management of AST, including conservative antibiotic treatment, incision and drainage, and open surgery combined with partial thyroidectomy. The choice of treatment is generally based on the state of AST. For instance, treatment with an antibiotic alone can be effective in managing AST without abscess formation or with small abscess ( 2 ). This is generally found in patients with short course of disease (e.g. the recurrence of case 5 in the present study). Surgical drainage, which was traditionally used to treat AST, has recently been replaced by needle aspiration ( 9 ), with research showing that this combination of aspiration and catheter drainage can be successful in the treatment of AST with large and liquefied abscess, and severe inflammatory response ( 8 ). This condition is common in patients with long course (e.g. case 2, 4 and 7). Nevertheless, the available papers are mostly case reports. In the present study, aspiration with or without lavage has many advantages over surgical incision and drainage, including rapid relief of clinical symptoms, cost-effectiveness and minimal invasiveness. In addition, lavage may help to open the fistula channel of the PSF, making the diagnosis of PSF easier. In the current study, we found that aspiration alone can be performed in patients with abscess formation but without complete liquefaction, whereas aspiration combined with lavage can be performed in patients with abscess liquefaction. These results suggest that US-guided needle aspiration with or without lavage is effective in the management of AST.

**Figure 5:**
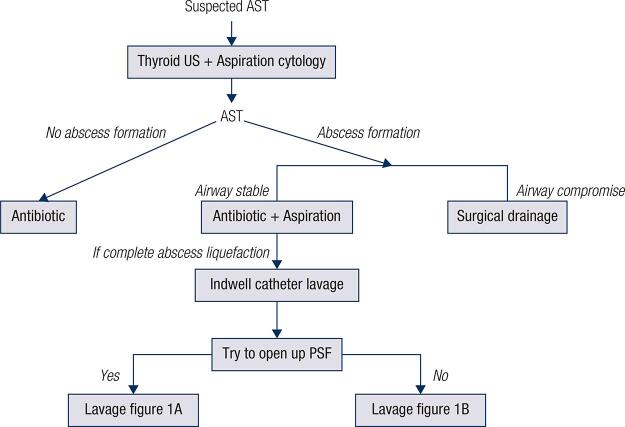
Management strategy for AST secondary to PSF. AST: acute suppurative thyroiditis; MRI: magnetic resonance imaging; PSF: pyriform sinus fistula; US: ultrasound.

Based on these results and the results of previous studies ( 2 , 21 ), we suggest that the management of AST should be based on the patient’s symptoms and signs and on the morphologic characteristics of AST, as seen on US images. We propose a management strategy for AST that will allow clinicians to determine the best course of action for each individual case ( Figure 5 ).

In this study, the diagnosis of PSF was based on imaging examinations, including esophagram, CT, MRI, and laryngoscopy. Laryngoscopy is increasingly being used to identify the internal opening of PSF, and it appears to be more sensitive than CT and esophagram ( 2 ). Nevertheless, there is currently no consensus regarding the optimal modality for the diagnosis of PSF ( 22 - 27 ) (Supplementary Table 2). Complicating the clinical picture, the sensitivity and ability of imaging examinations for the diagnosis of PSF vary depending on the stage of inflammation ( 22 , 25 , 26 ). One study demonstrated that the positive diagnostic rates for esophagram and CT are 89% and 20%, respectively, during the early or acute inflammatory stage and 97% and 54%, respectively, during the late inflammatory stage ( 27 ). These lower positive diagnostic rates during the early or acute inflammatory phase can be explained by swelling of the mucosa and adjacent tissue, which can cause partial or complete obstruction of the fistula tract. Similar to cases 4, 5 and 7, PSFs were difficult to find during acute inflammatory stage but higher positive rate by esophagram, CT, MRI, and laryngoscopy during the quiescent stage.

Furthermore, the present study suggests that the sensitivity and diagnostic ability of imaging examinations for identifying PSF are affected by many factors other than the stage of inflammation. Patient age and the size of the PSF are two vital factors. Younger patients generally have a larger fistula and fistula orifice, which simplifies the diagnosis of PSF. In this study, the large fistula in case 1 (patient age, 10 years) was identified on all imaging examinations performed during both stages, and the large fistula in case 5 (patient age, 8 years) was identified on laryngoscopy, esophagram, and CT during the quiescent stage. When the PSF is very fine (as usually occurs in adult patients), lavage may effectively open the PSF (cases 2 and 3), but the fistula orifice may then close after treatment, which complicates diagnosis during the quiescent stage (as was seen in case 3). The technique used in the examinations also affects their sensitivity and diagnostic ability. In this study, CT scan had negative results when it was performed immediately after injection of meglumine diatrizoate into the thyroid abscess, but had positive results when performed 2 h after injection (as in case 4). Each imaging examination has its own advantages and disadvantages, and there is no absolute comparability. Laryngoscopy can just observe the fistula orifice, whereas CT is effective in identifying the fistula. MRI has advantage in the location of cervical inflammation and may detect gases. Therefore, selecting the appropriate imaging modalities can be more effective according to the inflammation state, age, the size of the PSF, and patient tolerance. For the evaluation of fine PSF in the quiescent stage, ordinary CT scans are less sensitive. Considering the potential presence of a fistula, we explored an effective method of finding PSF: CT was performed immediately after oral barium used (cases 1, 4, 5, and 6 were all positive), then, considering the large granule of barium, we performed CT after oral meglumine diatrizoate and finding the indetectable fistula (case 2, 7). Therefore, we propose the use of CT immediately after oral meglumine diatrizoate as it may improve diagnostic sensitivity for identifying fine fistula.

PSF should be treated to reduce the recurrence rate of AST. If a PSF is present during follow-up, surgical fistulectomy or nonsurgical obliteration of the fistula should be considered. In small case series, the use of endoscopic chemocauterization has been shown to be successful and with a lower risk of complications compared with surgical fistulectomy ( 2 ). In a study of five children with PSF who were treated with electrocautery/CO _2_ laser/silver nitrate cauterization, no recurrence of AST was seen over 25 months of follow-up ( 28 ). In another study of 11 patients with PSF who underwent endoscopic CO _2_ laser cauterization, all had an uneventful recovery and remained symptom-free over 11 to 35 months of follow-up ( 29 ). A study suggested that a spontaneous cure may be possible in some patients with a very fine fistula ( 4 ). In this study, all imaging examinations of case 3 were negative during the quiescent stage. The self-closing of the PSF was highly suspected due to her elder age and smaller fistula. This will be verified during long-term follow-up.

Limitations of the current study include its retrospective nature and the short observation time, which may have biased the results. Long-term follow-up are needed to validate our proposed management strategy for AST.

In conclusion, US-guided aspiration with or without lavage had a good treatment effect and without adverse events for the management of AST secondary to PSF. The diagnosis of PSF was based on imaging examinations and the sensitivity and diagnostic ability of those examinations were affected by many factors other than the stage of inflammation.

## Appendix

**Supplementary Table 1:** Patient demographics, clinical characteristic, and etiology

Case	Age/ Sex	AST	Misdiagnosis	Symptoms	Interval 1 (d) ^a^	Interval 2 (d) ^b^	WBC* 10 ^9^ /L	ESR (mm/h)	CRP (mg/l)	PCT (ng/mL)	TSH (μIU/mL)	FT3 (pmol/l)	FT4 (pmol/l)	Abscess Size (cm)	Aspiration pus amount
1	10/F	Primary	——	Anterior neck pain and swelling, fever	4	0	18.15	109	30.5	0.05	1.58	4.1	12.5	3.7*3.5	20 mL
2	21/F	Primary	SAT	Anterior neck pain and swelling, odynophagia, fever, dysphonia	20	1	15.84	80	109.8	0.05	0.02	5.2	25.6	4.8 × 3.2	28 mL
3	26/F	Primary	Pharyngitis	Anterior neck pain and swelling, odynophagia, fever	7	0	7.94	45	38.6	NP	0.08	6.9	24.6	4.2 × 2.2	15 mL
4	18/M	Primary	Pharyngitis	Anterior neck pain and swelling, odynophagia, fever, headache	20	0	14.09	95	226.8	0.10	0.02	3.8	23.1	8.6 × 4.3	36 mL
5	8/F	Primary	URI	Anterior neck pain and swelling, odynophagia, fever	11	0	17.47	105	148.4	0.11	1.88	4.4	23.7	4.6 × 3.0	10 mL
6 ^c^	15/F	Relapse	SAT	Anterior neck pain and swelling, fever	7	2	7.36	NP	80.7	0.05	NP	NP	NP	2.6 × 1.4	8 mL
7	20/M	Primary	Pharyngitis	Anterior neck pain and swelling, odynophagia, fever, dysphonia	21	0	15.7	80	201.3	0.14	0.02	3.3	16.6	7.7 × 3.7	45 mL

**Case**	**Cytology**		**Pus culture**	**Blood culture**	**Antibiotic types**		**Antibiotic dose**	**Aspiration**	**Lavage**	**Imaging**		**Follow-up (mo)**		**Outcome**	**Status of PSF**

1	Neutrophils		Streptococcus spp.	Negative	Ceftriaxone		2.0 g/d	1	0	US, esophagram, CT		29		Completely recovered	Excision
2	Lymphocytes and neutrophils		Streptococcus spp.	Negative	Clindamycin 3 days then Vancomycin + levofloxacin		1.8 g/d 2.0 g/d 0.4 g/d	2	2	US, CT, MRI, laryngoscopy, esophagram		21		Completely recovered	Stable
3	Neutrophils		Negative	NP	Amoxicillin sulbactam + levofloxacin		6.0 g/d 0.4 g/d	2	2	US, CT, laryngoscopy, esophagram, MRI		20		Completely recovered	Stable
4	Inflammatory cells and debris		Streptococcus spp.	Negative	Amoxicillin sulbactam + ornidazole		9.0 g/d 0.5 g/d	1	6	US, CT, MRI, esophagram, laryngoscopy		17		Completely recovered	Stable
5	Neutrophils		Negative	NP	Cefotiam		2.0 g/d	1	0	US, CT, MRI, esophagram, laryngoscopy		20		Completely recovered; relapse at 9 months	Stable
6 ^c^	Neutrophils		Negative	Negative	Amoxicillin sulbactam +ornidazole		9.0 g/d 1.0 g/d	1	0	US, esophagram, CT, laryngoscopy		18		Completely recovered	Stable
7	Inflammatory cells and debris		Streptococcus spp.	Negative	Mezlocillin sulbactam		10.25 g/d	2	2	US, CT, MRI, esophagram, laryngoscopy		3		Completely recovered	Stable

AST: acute suppuration thyroiditis; CT: computed tomography; MRI: magnetic resonance imaging; NP: not performed; PSF: pyriform sinus fistula; US: ultrasound; WBC: white blood cell; ESR: erythrocyte sedimentation rate; CRP: C-reactive protein; PCT: procalcitonin; TSH: thyroid-stimulating hormone; SAT: subacute thyroiditis; URI: upper respiratory infection.

^a^ Interval 1: interval between the onset of symptoms and initial diagnosis of AST.

^b^ Interval 2: interval between the diagnosis of AST and aspiration.

^c^ This patient was diagnosed with AST 1 year previously and recovered with conservative treatment.

## Appendix

**Supplementary Table 2:** Rates of AST diagnosis for various imaging modalities in previous studies

Study	Year	Study type	Patients, n	Population	Period	Laryngoscopy	Esophagram	CT	MRI
Nicoucar et al. (24)	2009	Review	526	Children	N/A	45%	74%	N/A	N/A
Nicoucar et al. (25)	2010	Review	202	Children	N/A	82%	81%	49%	84%
Kim et al. (22)	2000	Original study	18	Patients aged ≤ 14 years (n = 11); patients aged > 14 years (n = 7)	N/A	100%	50%	80%	N/A
Parida et al. (23)	2014	Original study	17	Children	N/A	100%	88.2%	N/A	N/A
Liang et al. (26)	2016	Original study	63	Patients aged ≤ 14 years (n = 26); patients aged > 14 years (n = 37)	N/A	N/A	74.6%	85.7%	84.4%
Masuoka et al. (27)	2011	Original study	60	N/A	Early Late	N/A N/A	89% 97%	20% 54%	N/A N/A

CT: computed tomography; MRI: magnetic resonance imaging; N/A: not available.
